# Completion Audit of Inpatient Glasgow Anti-Psychotic Side-Effect Scale (GASS) Forms

**DOI:** 10.1192/bjo.2024.636

**Published:** 2024-08-01

**Authors:** Andrew Tait, Eugene Wong

**Affiliations:** University Hospital Hairmyres, East Kilbride, United Kingdom

## Abstract

**Aims:**

Side-effects are a recognised burden of all medications and are linked to poor compliance. In psychiatry, poor compliance can result in a relapse and significant deterioration in mental health. This has an impact on both the patient and the wider healthcare system. It has been speculated that if patients had more control/recognition of side-effects, compliance would increase.

GASS is a self-rating scale for side-effects of antipsychotic medication. It has the added effect of being able to stratify side-effects by their severity and biological system involved (Central Nervous System (Sedating) effects, Neurological (Movement) disorders, Anticholinergic, Gastrointestinal and Endocrine). The form consists of 22 questions with a scoring sheet attached to the reverse. Symptoms are graded by frequency and patient's perceived burden.

The British National Formulary has ‘minimum standards’ expected. These are designed to create a standardised approach to side-effect reviewing, encouraging a proactive reviewing process. These are meant to take place: After initiation and dose titration, at 3 months and annually thereafter. The National Institute for Clinical Excellence Guideline CG178 and the Scottish Intercollegiate Guidelines Network (Guideline-131) both advocate this standardised approach with the gold standard adding a review at 1 month.

The aim for this project was to audit the current completion rate of GASS forms in inpatient wards. The secondary aim was to improve completion rates after intervention.

**Methods:**

1. Search case notes and extrapolate data to Microsoft excel.
2. Review data and identify challenges perceived from staff.
3. Implement changes targeting highlighted challenges.
  1. Present at ward QI meetings.
  2. Create & discuss Infographic for staff.
  3. Highlight role/importance of forms and usefulness to clinicians.
4. Re-audit after 2 months.

**Results:**

Initial results found a completion rate of 7% across both wards reviewed (n = 41). Within this, 1 form was actually valid. One of the wards had no completed forms. The post-intervention group had fewer patients involved (n = 35), but an increased number of completed forms. Completion rate had risen from 7% to 26% (3–9 patients). Within this, the valid forms had increased from 1 to 4.

**Conclusion:**

There was a clear impact on completion rate after initial interventions. The sub-optimal increase in completion highlighted the ongoing need for further input to improve completion rates.

This was a small, local audit of patients in an acute inpatient psychiatric ward. There was a recognised limitation on the number of patients in the study and acuity of some patient's illness, preventing completion.

